# Development and internal validation of PI-RADs v2-based model for clinically significant prostate cancer

**DOI:** 10.1186/s12957-018-1367-9

**Published:** 2018-06-01

**Authors:** Yu Zhang, Na Zeng, Yi Chen Zhu, Yang Xin Rui Huang, Qiang Guo, Ye Tian

**Affiliations:** 1 Department of Urology, Beijing Friendship Hospital, Capital Medical University, No. 95, Yongan Road, Xicheng District, Beijing, People’s Republic of China; 2 National Clinical Research Center for Digestive Diseases, Beijing Friendship Hospital, Capital Medical University, No. 95, Yongan Road, Xicheng District, Beijing, People’s Republic of China

**Keywords:** PI-RADs v2, Prostate cancer, Model

## Abstract

**Background:**

Our objective is to build a model based on Prostate Imaging Reporting and Data System version 2 (PI-RADs v2) and assess its accuracy by internal validation.

**Methods:**

Patients who took prostate biopsy from 2014 to 2015 were retrospectively collected to compose training cohort according to the inclusion criteria and patients in 2016 composing validation cohort. Diagnostic performance was evaluated by analyzing the area under the curve (AUC), calibration curves, and decision curves.

**Results:**

Of the 441 patients involved, the clinically significant prostate cancer (csPCa) detection rate were 40.6% (114/281) and 43.8% (70/160) in the training and validation cohort, respectively. Meanwhile, PCa detection rate were 50.2% (141/281) and 53.8% (86/160). Age, prostate-specific antigen density (PSAD)*10 and PI-RADs v2 score composed the model for PCa (model 1) and csPCa (model 2). The area under the curve of models 1 and 2 was 0.870 (95% CI 0.827–0.912) and 0.753 (95% CI 0.717–0.828) in the training cohort, while 0.845 (95% CI 0.786–0.904) and 0.834 (95% CI 0.787–0.882) in the validation cohort. Both models illustrated good calibration, and decision curve analyses showed good performance in predicting PCa or csPCa when the threshold was 0.35 or above.

**Conclusions:**

The model based on age, PSAD*10 and PI-RADs v2 score showed internally validated high predictive value for both PCa and csPCa. It could be used to improve the diagnostic performance of suspicious PCa.

## Background

Prostate cancer (PCa) ranks as the second most common malignancy in male population and has been the second leading cause of cancer-related mortality in Western men [1]. Though the high morbidity and mortality exist, advancements in the early diagnosis attribute much to the improvement of life expectancy. The conventional screening pathway mainly emphasized elevated prostate-specific antigen (PSA) and abnormal digital rectal examination (DRE). However, both the sensitivity and specificity were found to be suboptimal and insufficient for early detection [2].

Multiparametric magnetic resonance imaging (mpMRI) enjoys priority in visualization of prostate due to its high soft-tissue contrast, high resolution, and simultaneous image functional parameters [3]. To set standardized reporting and propose criteria for interpreting data of mpMRI, the European Society of Urogenital Radiology (ESUR) published a reporting system termed Prostate Imaging Reporting and Data System version 1 (PI-RADs v1) in 2012, which was based on four MRI sequences (T2-weighted imaging (T2WI), diffusion-weighted imaging (DWI), dynamic contrast enhanced MRI (DCE-MRI), and MR spectroscopy) [4]. Though PI-RADS v1 system has been validated the accuracy and reproducibility, however, it was not specified exactly how to combine each MRI sequence to derive an overall category assessment, which resulted in confusion in its application. To address this issue, the ESUR and American College of Radiology agreed on the improved PI-RADS version 2 (PI-RADSv2) released online in 2014 [5]. The intended clinical application of PI-RADS v2 is for the diagnostic evaluation as well as risk assessment, and the assessment category of transition zone lesions is mainly determined by the T2WI score while that of peripheral zone lesions is defined by the DWI score [6]. Several studies have validated the high sensitivity and specificity of PI-RADs v2 in diagnosis of prostate cancer [4–7], and updated PI-RADSv2 shows significant improvement compared with the original Prostate Imaging Reporting and Data System (PIRADS) v1.

There were several risk calculators for PCa, such as European Randomized Study for Screening of Prostate Cancer Risk Calculator (ERSPC-RC), Prostate Cancer Prevention Trial Risk Calculator (PCPT-RC), and Chinese Prostate Cancer Consortium Risk Calculator (CPCC-RC) [8]. The validity of all of the above has been validated in previous studies. However, none of them is composed of PI-RADs v2. The primary objective of this study is to build a model based on PI-RADs v2 and assess its accuracy by internal validation.

## Methods

### Study population and data collection

Five hundred forty-three men with suspicion of PCa (elevated PSA levels and/or suspicious DRE) who were biopsy-naive were collected and registered into a reprospective database after the approval of Ethical Committee of Beijing Friendship hospital. Transrectal ultrasound (TRUS)-guided 24-core biopsy was given from January 2014 to December 2016. The exclusive criteria were as follows: patients with urinary tract infection, urinary retention, or consistent catheterization within the past 2 weeks [1]; patients who received 5α-reductase inhibitors within the last 2 months [2]; those aged older than 90 years old or who had PSA level greater than 100 ng/ml [3]; those with previous history of transurethral resection of prostate (TURP) [4]; and patients without recording of PSA value, age, or MRI-measured prostate volume (PV) [5]. After that, a total of 441 patients were included, which were composed of 281 patients in the training cohort from 2014 to 2015 and 160 patients in the validation cohort from 2016. All of them received mpMRI before biopsy.

### mpMRI protocol

The prostate mpMRI was performed at 3 Tesla (T) as recommended [5]. The acquisition protocol included T2WI, T1WI, DWI with apparent diffusion coefficient map (ADC), and DCE sequences and calculated *b* value of 1000 or above. Each sequence used a five-point assessment scale (except for DCE) which graded the level of suspicion for the presence of PCa from 1 to 5 (very low to very high) [5]. The dominance sequence is used according to zonal anatomy. DWI was the primary determining sequence of the peripheral zone (PZ), while T2WI was mainly for the transitional zone (TZ). DCE had limited contribution as merely presence and absence of early focal enhancement when T2W and DWI were of adequate diagnostic quality. However, it played a supporting role in the indeterminate category 3 PZ lesions. A urologist who was experienced with PI-RADs v2 and blinded to histopathology as well as clinical data reviewed all the images and performed scoring.

### Histopathological analysis

The TRUS-guided systematic biopsy of 24-needle cores (20 cores in PZ and 4 cores in TZ) were performed within 3 months after MRI. A uropathologist with more than 20 years in urological pathology revised the histopathology results and assigned Gleason scores. The clinically significant prostate cancer (csPCa) was defined as Gleason score (GS ≥ 4 + 3 or 3 + 4 with PSA > 10 ng/ml, > 3 biopsy cores positive, or at least one biopsy core with > 50% involvement), according to Epstein criteria [9].

### Statistical analysis

Sensitivity, specificity, positive predictive value (PPV), and negative predictive value (NPV) with 95% confidence interval (CI) were calculated for diagnostic accuracy of PI-RADs v2 in contrast to histological findings. Independent *T* test and chi-square test were performed to determine significant differences in baseline characteristics. Univariate and multivariate logistic regression was performed to explore the relationship between variables and results (PCa or csPCa). Multivariate logistic regression model for predicting PCa and csCa was constructed. The diagnostic performance of the model was assessed by receiver operating curves (ROC) and comparing diagnostic accuracy in validation cohort. Calibration curves were used to assess the extent of over- or underestimation of the models. Decision curves were applied to determine the clinical net benefit derived from the use of the model. The area under the curve (AUC) was applied for the assessment of the accuracy. *P* value less than 0.05 was considered to indicate a statistically significant. All analyses were performed with SPSS software (Version 21.0. IBM), and R version 3.0.0 and the figures were painted using GraphPad Prism 5.

## Results

### Characteristics and biopsy outcomes

All the enrolled people were of yellow race. There are 281 patients and 160 patients in the development cohort and validation cohort, respectively. One hundred forty-one patients (50.1%) and 86 patients (53.8%) were diagnosed PCa in the development cohort and validation cohort (*P* = 0.06), respectively. While 114 patients (40.6%) and 70 (43.8%) patients were diagnosed csPCa in the two cohorts (*P* = 0.12). In the training cohort, 94 patients were diagnosed with csPCa for GS ≥ 4 + 3 and 20 patients for GS = 3 + 4 with PSA > 10 ng/ml, > 3 biopsy cores positive, or at least one biopsy core with > 50%. In the validation cohort, 66 patients were diagnosed with csPCa for GS ≥ 4 + 3 and 4 patients for GS = 3 + 4 with PSA > 10 ng/ml, > 3 biopsy cores positive, or at least one biopsy core with > 50%.

The median PSA level was 11.9 ng/ml (interquartile range; IQR 6.8–25.5) and 11.7 ng/ml (IQR 7.4–26.7) in the development cohort and the validation cohort, median age was 70 (IQR 63–77 years) and 65 (IQR 62–75 years), median PV was 49.0 ml (IQR 35–73.4) and 43.9 ml (IQR 36.5–59.4), and median prostate-specific antigen density (PSAD) was 0.23 (IQR 0.12–0.55) and 0.24 (IQR 0.13–0.43), respectively. No differences were observed with regard to age, PSA, PV, PSAD (all *p* > 0.05). When the PI-RADS v2 score was 3, significant difference was observed between the two cohorts. Table 1 showed the baseline characteristics between the two groups.

**Table 1:** Baseline characteristics of 281 patients in development cohort and 160 patients in validation cohort (chi-square test and independent *T* test)

Origin of cohort	Training cohort	Validation cohort	*P* value
No. of Pts	281	160	
No. of PCa (%)	141 (50.2)	86 (53.8)	0.68
No. of csPCa (%)	114 (40.6)	70 (43.8)	0.68
Age, median (IQR),	70 (63–77)	65 (62–75)	0.08
PSA, median (IQR), ng/ml	11.9 (6.8–25.5)	11.7 (7.4–26.7)	0.24
PV, median (IQR), ml	49.0 (35–73.4)	43.9 (36.5–59.4)	0.16
PSAD, median (IQR), ng/ml^2^	0.23 (0.12–0.55)	0.24 (0.13–0.43)	0.32
PIRADS v2			
1	6 (2.1)	0 (0.0)	0.07
2	41 (14.6)	37 (23.1)	0.06
3	75 (26.7)	22 (13.8)	0.01
4	83 (29.5)	50 (31.3)	0.78
5	76 (27.0)	51 (31.9)	0.43

*Pts* patients, *No*. number, *PCa* prostate cancer, *csPCa* clinically significant prostate cancer, *PSA* prostate-specific antigen, *IQR* interquartile range, *PV* prostate volume, *PSAD* prostate-specific antigen density, *PIRADS v2* Prostate Imaging Reporting and Data System version 2

### Efficiency of PI-RADS v2 alone in diagnosis of PCa and csPCa

PI-RADs v2 was proved to be 76.6% sensitive and 83.6% specific with positive predictive value 67.9% and negative predictive value 73% when used alone for diagnosis of PCa. However, when assessing the diagnostic performance of PI-RADs v2 for csPCa, the sensitivity and specificity were 85.9 and 63.5%, respectively, with positive predictive value 61.6% and negative predictive value 86.9%. Table 2 showed the diagnostic performance of PI-RADs v2 alone.

**Table 2:** Diagnostic performance of PIRADS v2 alone for PCa and csPCa

	PCa				csPCa			
	PI-RADs v2 (%)	Age (%)	PSAD (%)	Model 1 (%)	PI-RADs v2 (%)	Age (%)	PSAD (%)	Model 2 (%)
Sensitivity	76.6	51.8	83	85.8	85.9	78.1	78.9	87.5
Specifivity	83.6	69.2	48.9	67.9	63.5	65.3	43.7	67.1
Positive predictive value	67.9	49.7	62.2	72.9	61.6	60.5	48.9	63.1
Negative predictive value	73	69.2	73.9	82.6	86.9	81.3	72.3	84.8

### Construction of prediction models

**Fig. 1:**
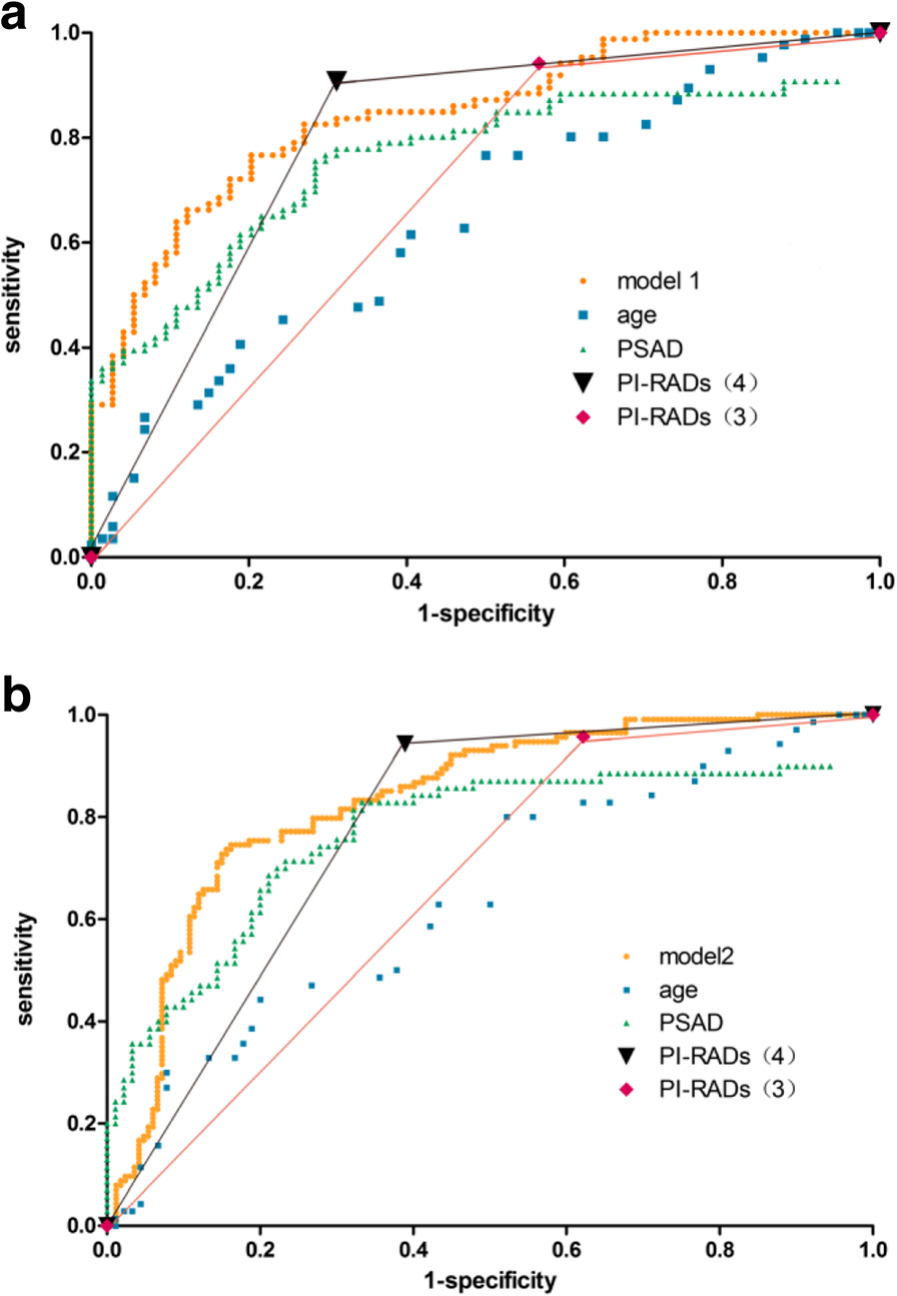
The ROC curves of two prediction models in validation cohort. Model 1 (**a**) and model 2 (**b**)

At univariate analysis, all variables represented independent predictors of PCa and csPCa (all *p* < 0.05, Table 3). Further multivariate analysis showed that only age, PSAD*10, and PI-RADs v2 score were significantly associated with biopsy results (PCa or csPCa). So, these parameters were entered into the prediction models which stood for PCa (model 1) and csPCa (model 2). In the development cohort, model 1 achieved an area under the curve (AUC) of 0.870 (95% CI 0.827–0.912) and the AUC was 0.753 (95% CI 0.678–0.828) for model 2 predicting csPCa. In the validation cohort, the AUC was 0.845 (95% CI 0.786–0.904) in predicting PCa and 0.834 (95% CI 0.787–0.882) in predicting csPCa, Table 4, Fig. 1. The diagnostic performance of the two models was significantly better than each single variable (*p* < 0.05), showed in Table 4.

**Table 3:** Univariate and multivariate logistic regression predicting PCa and csPCa (logistic regression analysis)

	PCa				csPCa			
	Univariate analysis		Multivariate analysis		Univariate analysis		Multivariate analysis	
	OR (95% CI)	*p* value	OR (95% CI)	*p* value	OR (95% CI)	*p* value	OR (95% CI)	*p* value
PSA	1.06 (1.04–1.09)	< 0.001	1.04 (0.94–1.08)	0.14	1.02 (1.01–1.03)	< 0.001	1.00 (0.98–1.02)	0.63
PV	0.99 (0.98–1.00)	0.003	0.97 (0.93–1.04)	0.11	1.00 (0.99–1.01)	0.76	0.99 (0.98–1.00)	0.12
PSAD*10	1.55 (1.34–1.79)	< 0.001	1.32 (1.01–1.72)	0.04	1.07 (1.03–1.11)	0.001	1.01 (1.00–1.02)	0.01
Age	1.11 (1.08–1.16)	< 0.001	1.15 (1.10–1.21)	< 0.001	1.17 (1.13–1.22)	< 0.001	1.18 (1.12–1.23)	< 0.001
PIRADS	1.18 (1.04–1.31)	0.03	2.22 (1.07–4.63)	0.03	1.29 (1.03–1.77)	0.01	2.54 (1.25–5.17)	0.01

*PCa* prostate cancer, *csPCa* clinically significant prostate cancer; prostate-specific antigen, PSAD*10 prostate-specific antigen density*10, *PIRADS v2* Prostate Imaging Reporting and Data System version 2, *PV* prostate volume, *OR* odds ratio

**Table 4:** Areas under the curve of the calculated variables of model predicting the presence of PCa or csPCa in the validation cohort (chi-square test)

	PCa	*P* value (compared to model)	csPCa	*P* value (compared to model)
Predictors	AUC (95% CI)		AUC (95% CI)	
Model	0.845 (0.786–0.904)		0.834 (0.787–0.882)	
Age	0.650 (0.566–0.735)	0.001	0.646 (0.560–0.732)	0.002
PSAD*10	0.762 (0.686–0.837)	< 0.001	0.769 (0.689–0.848)	< 0.001
PIRADs v2 (threshold 4)	0.798 (0.725–0.871)	< 0.001	0.777 (0.704–0.850)	< 0.001
PIRADs v2 (threshold 3)	0.687 (0.602–0.772)	< 0.001	0.667 (0.585–0.750)	< 0.001

*PCa* prostate cancer, *csPCa* clinically significant prostate cancer; prostate-specific antigen, *PSAD* prostate-specific antigen density, *PIRADS v2* Prostate Imaging Reporting and Data System version 2, *AUC* area under the curve

### Calibration and decision curves of the models

**Fig. 2:**
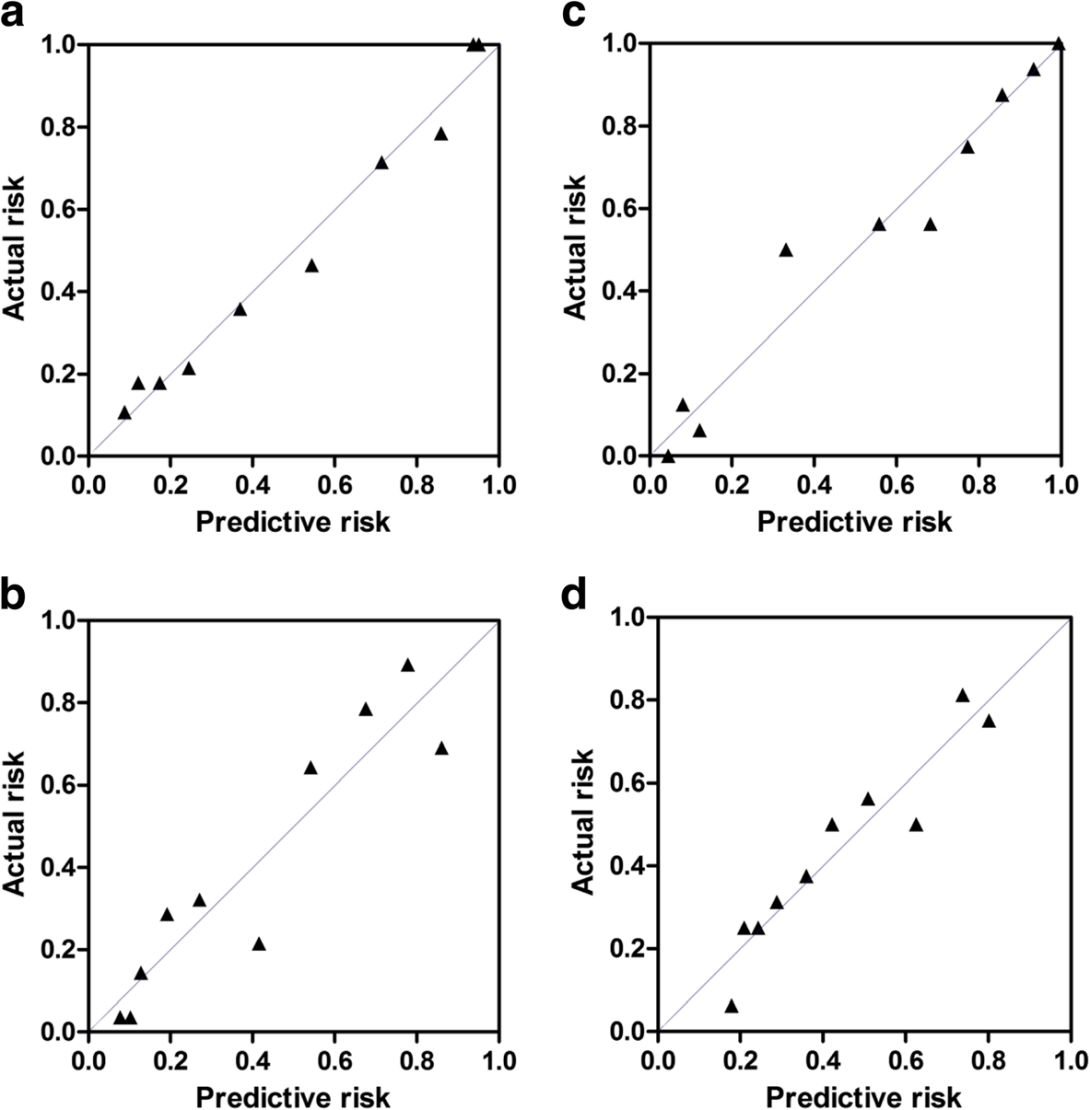
Calibration curves for the prediction models. **a** Model 1 in the development cohort, **b** model 2 in the development cohort, **c** model 2 in the validation cohort, and **d** model 2 in the validation cohort. The 45° dotted line represents perfect prediction by ideal model

**Fig. 3:**
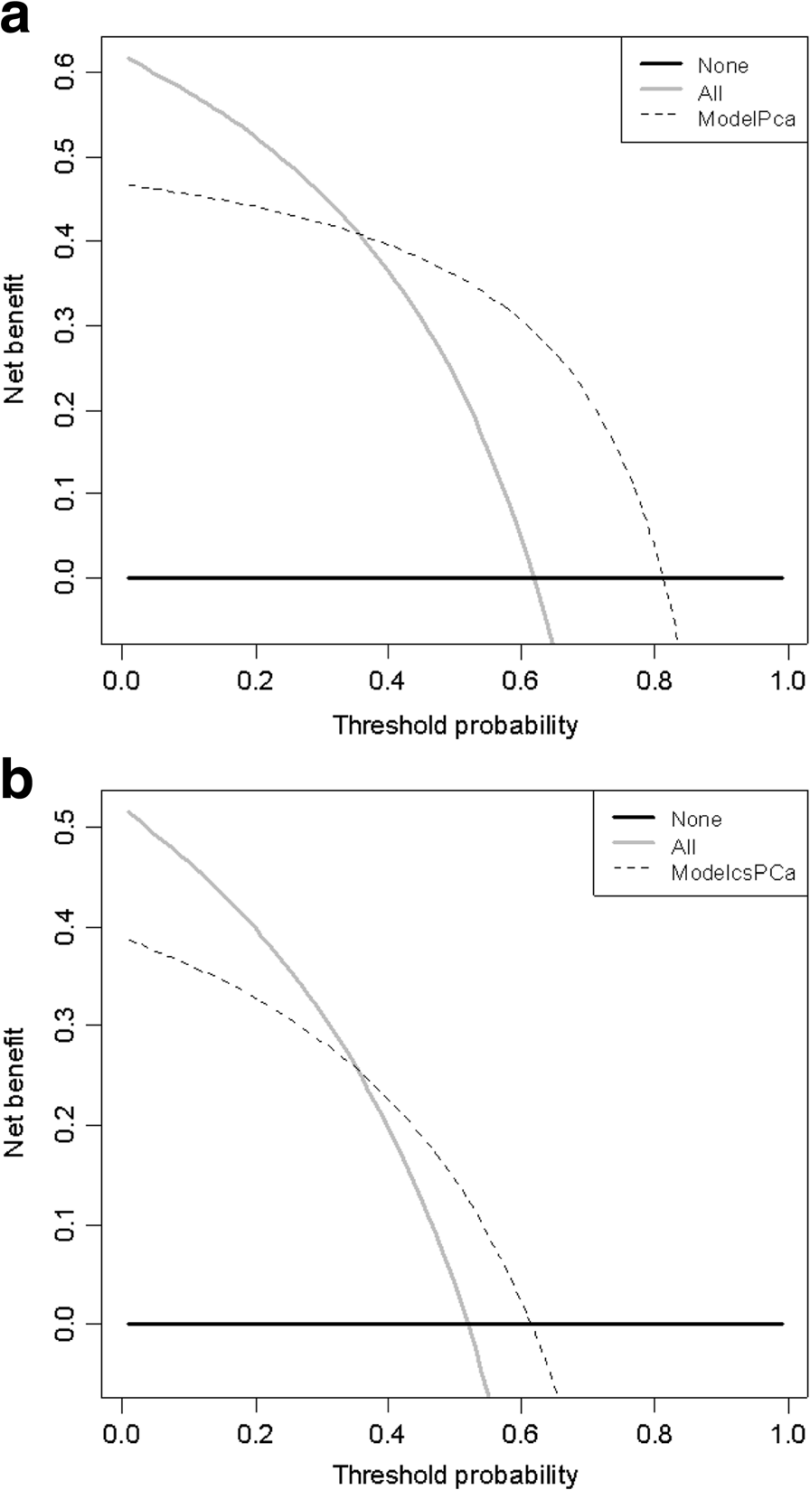
Decision curve analysis demonstrating the net benefit associated with the use of the model 1 **a** and model 2 **b**. None means “treat none,” and all means “treat all.” Model PCa (csPCa) means “treat those with PCa (or csPCa) predicted by model”

Figure 2 displayed calibration curves of both models 1 and 2. On each calibration plot, the predicted risk of the model was represented on the *x* axis and the actual risk of biopsy-proven PCa or csPCa is represented on the *y* axis. Within the internal validation cohort, equally excellent calibration curves were observed. Decision curves showed that the models resulted in a higher net benefit when the threshold probabilities was 0.35 or above for both csPCa and PCa. (Fig. 3).

## Discussion

It is showed in our study that PI-RADs v2 performed a higher sensitivity and negative predictive value when assessing the detection of csPCa than PCa. And the validation provided evidence supporting both models 1 and 2 that were based on PI-RADs v2, age, and PSAD*10 in predicting csPCa and PCa. The performance of the two models was significantly better than each single variable. Calibration properties were good in patients with PCa and csPCa. These findings were further supported by a decision curve analysis. Several recent studies focusing on the validity of PI-RADs v2 scoring system in detection of csPCa or PCa have validated the diagnostic performance. Though the outcome varied among studies, PI-RADs v2 was proved to have high accuracy for predicting csPCa [2–4, 6, 7]. One of these studies [6] resulted in AUC of PI-RADs v2-only of 0.83 in PCa and 0.91 in csPCa, which was higher than ours. A possible reason for this might be that our AUC analysis was based on pathological results and experimental examinations, while they made analysis basing on lesions. In their study, patients with suspicious findings, at least one lesion with a PI-RADS v1 assessment category of ≥3, were selected for biopsy and included in the cohort. And that made a great difference. Besides, targeted in-bore MR-guided biopsy helped find more csPCa comparing to our TRUS-guided systematic biopsy of 24-needle cores.

Another study [10] has shown the accurate prediction of PI-RADs v2 based model for high-grade PCa, which also comprised PI-RADs v2, age, and PSAD. On comparing that work to the present study, the model in the present study enrolled more patients (441 versus 247) and showed a lower AUC (83 versus 86%). The reason for this might be that their biopsy was based on targeted lesions whose PI-RADS v1 sum score > 9, and this led to high detection of csPCa. Clinically significant PCa in the present study was defined as GS ≥4 + 3 or 3 + 4 with PSA > 10 ng/ml, > 3 biopsy cores positive, or at least 1 biopsy core with > 50% involvement. Comparing to definition of GS ≥ 7 in their study, less csPCa were observed in our cohort.

There are several predicting tools that have been increasingly developed and validated for use in the PCa screening, such as the European Randomized Study for Screening of Prostate Cancer Risk Calculator (ERSPC-RC) and Prostate Cancer Prevention Trial Risk Calculator (PCPT-RC). Though some variables were found, they were mainly based on age, family history, PSA level, DRE, PV, and previous biopsy status [11, 12]. The Chinese Prostate Cancer Consortium Risk Calculator (CPCC-RC) performed better in decision making of prostate biopsy in Chinese or in other Asian populations included PSA, PV, age, free PSA ration, and DRE but did not involve family history or prior biopsy [8]. However, all the risk calculators above did not take the weight of mpMRI into account. The model established in this study highlighted the dominance of PI-RADS v2 scoring in prediction and showed an AUC of 0.845 (0.786–0.904) for PCa and 0.834 (0.787–0.882) for csPCa in validation cohort, which outperformed the CPCC-RC (AUC 0.801 and 0.826).

The relationship between PSA screening and PCa have been evaluated in both Chinese and Western populations, though it differs importantly between them [13, 14]. A previous study [1] carried out a comprehensive epidemiological analysis of global PCa incidence and mortality using high-quality data. China has the increasing incidence and staple mortality compared to western countries. Prostate volume was proved to be higher in Chinese compared to western population, which could theoretically lead to a higher PSA value and miss PCa at biopsy [14]. PSAD, which could eliminate the influence of PV on PSA, was proved to be a significant predictor for PI-RADs 3–5 lesions [15, 16]. Also, a recent study [17] has validated the incremental value of PSAD in combination with PI-RADS for the accuracy of PCa screening and showed that the NPV of PI-RADS could be improved by inclusion of PSAD and unnecessary biopsies could be reduced. Even for PCa men on active surveillance, combining PSAD and PIRADS score could predict upstaging when PIRADS score is > = 3 with PSAD > 0.15 [18]. We entered PSAD into the model, and it resulted in an excellent diagnostic performance.

In view of the fact that the benefit of mpMRI is becoming an increasingly important aspect of urologic practice [19], there are several reasons that the development of this model should be favored. First of all, it combines PI-RADs v2 with clinical factors PSAD and age, resulting in good clinical performance among both urologists and radiologists. Though moderate inconsistence still exists among the interobserver agreements, PI-RADs v2 reduce variability in imaging by establishing guidelines, summarizing suspicion levels, and standardizing reports. Clinical urologists could improve the diagnostic ability by learning the diagnostic process of PI-RADS v2. Secondly, all patients included in the study received 24-core systematic TRUS-guided biopsy, and the impact on tumor detection of different biopsy methods could be avoided. TRUS-guided systematic biopsy was validated to have similar overall detection compared to MRI-targeted Biopsy or MRI-TRUS fusion biopsy [20, 21], though the detection rate of csPCa might be lower. Last but not the least, this model included only three variables and made it simplified and applied for not only urologists but also radiologists, which was different from previous models.

There are several limitations of this study that should be noticed. The main limitation is a retrospective single-center design, and prospective multicenter external validation should be required to validate its accuracy better. Besides, our outcomes were got according to biopsy-proven Gleason score but not post-prostatectomy pathological grading, which may result in a lower diagnosis quantity of csPCa and make the predictive accuracy of the model be underestimated [22, 23]. Furthermore, we did not enter DRE which was previously proved even a better predictor than PSA into the model, because we wanted the model as objective as possible. And DRE was often performed by resident physicians in our center, which led to a wide difference when it came to the results positive or negative.

We recommend a further study on how would the model performed if we take PI-RADS v2 score 3 as the threshold rather than 4 in current study. And whether this model could be used to assess the diagnostic concordance of csPCa between biopsy results and post-prostatectomy pathological results will be explored in our next study.

## Conclusion

The model based on age, PSAD, and PI-RADs v2 score showed internally validated high predictive value for both PCa and csPCa. It could be used to improve the diagnostic performance of suspicious PCa. However, further multicenter external validation should be performed for its wide application.

## Abbreviations

csPCa: Clinically significant prostate cancer
DRE: Digital rectal examination
mpMRI: Multiparametric magnetic resonance imaging
PCa: Prostate cancer
PI-RADs: Prostate Imaging Reporting and Data System
PSA: Prostate-specific antigen
PSAD: Prostate-specific antigen density
PV: Prostate volume
TRUS: Transrectal ultrasound

## References

[1] Wong MC, Goggins WB, Wang HH, Fung FD, Leung C, Wong SY (2016). Global incidence and mortality for prostate cancer: analysis of temporal patterns and trends in 36 countries. Eur Urol.

[2] Gershman B, Van Houten HK, Herrin J, Moreira DM, Kim SP, Shah ND, Karnes RJ. Impact of Prostate-specific Antigen (PSA) Screening Trials and Revised PSA Screening Guidelines on rates of prostate biopsy and postbiopsy complications. Eur Urol. 2017;(57):55–65. 10.1016/j.eururo.2016.03.015 26995328

[3] Futterer JJ, Briganti A, De Visschere P, Emberton M, Giannarini G, Kirkham A (2015). Can clinically significant prostate cancer be detected with multiparametric magnetic resonance imaging? A systematic review of the literature. Eur Urol.

[4] Woo S, Suh CH, Kim SY, Cho JY, Kim SH. Diagnostic performance of prostate imaging reporting and data system version 2 for detection of prostate cancer: a systematic review and diagnostic meta-analysis. Eur Urol. 2017;72(2):177-88. 10.1016/j.eururo.2017.01.042 28196723

[5] Barentsz JO, Weinreb JC, Verma S, Thoeny HC, Tempany CM, Shtern F (2016). Synopsis of the PI-RADS v2 guidelines for multiparametric prostate magnetic resonance imaging and recommendations for use. Eur Urol.

[6] Kasel-Seibert M, Lehmann T, Aschenbach R, Guettler FV, Abubrig M, Grimm MO (2016). Assessment of PI-RADS v2 for the detection of prostate cancer. Eur J Radiol.

[7] Baldisserotto M, Neto EJ, Carvalhal G, de Toledo AF, de Almeida CM, Cairoli CE (2016). Validation of PI-RADS v.2 for prostate cancer diagnosis with MRI at 3T using an external phased-array coil. J Magn Reson Imaging.

[8] Chen R, Xie L, Xue W, Ye Z, Ma L, Gao X (2016). Development and external multicenter validation of Chinese prostate cancer consortium prostate cancer risk calculator for initial prostate biopsy. Urol Oncol.

[9] Ploussard G, Epstein JI, Montironi R, Carroll PR, Wirth M, Grimm MO (2011). The contemporary concept of significant versus insignificant prostate cancer. Eur Urol.

[10] Niu X, He W, Zhang Y, Das SK, Li J, Xiong Y, Wang YH. Developing a new PI-RADS v2-based nomogram for forecasting high-grade prostate cancer. Clin Radiol. 2017;72(6):458-64. 10.1016/j.crad.2016.12.005 28069159

[11] Ankerst DP, Hoefler J, Bock S, Goodman PJ, Vickers A, Hernandez J (2014). Prostate cancer prevention trial risk calculator 2.0 for the prediction of low- vs high-grade prostate cancer. Urology.

[12] Braun K, Sjoberg DD, Vickers AJ, Lilja H, Bjartell AS (2016). A four-kallikrein panel predicts high-grade cancer on biopsy: independent validation in a community cohort. Eur Urol.

[13] Gershman B, Van Houten HK, Herrin J, Moreira DM, Kim SP, Shah ND (2017). Impact of prostate-specific antigen (PSA) screening trials and revised PSA screening guidelines on rates of prostate biopsy and postbiopsy complications. Eur Urol.

[14] Chen R, Sjoberg DD, Huang Y, Xie L, Zhou L, He D (2017). Prostate specific antigen and prostate cancer in Chinese men undergoing initial prostate biopsies compared with Western cohorts. J Urol.

[15] Wegelin O, Van Melick H, Somford D, Bosch R, Kummer A, Vreuls W (2016). Clinical predictors of PIRADS ≥3 lesions on MP-MRI in patients with negative prior prostate biopsies and a persisting clinical suspicion on prostate cancer. Eur Urol Suppl.

[16] Truong M, Frye T, Lam D, Park JH, Wang B, Feng C (2017). Mp08-14 development and validation of a nomogram for predicting Pirads 4-5 lesions on multiparametric prostate MRI. J Urol.

[17] Distler FA, Radtke JP, Bonekamp D, Kesch C, Schlemmer HP, Wieczorek K, et al. The value of PSA density in combination with PI-RADS for the accuracy of prostate cancer prediction. J Urol. 2017;198(3):575-82. 10.1016/j.juro.2017.03.130 28373135

[18] Van Kuiken M, Blackwell RH, Bisanz B, Yacoub J, Goldberg A, Shea S (2017). Pd55-09 role of Mpmri Psa density and Pirads score in predicting upstaging in men on active surveillance. J Urol.

[19] Zhao C, Gao G, Fang D, Li F, Yang X, Wang H (2016). The efficiency of multiparametric magnetic resonance imaging (mpMRI) using PI-RADS version 2 in the diagnosis of clinically significant prostate cancer. Clin Imaging.

[20] Baco E, Rud E, Eri LM, Moen G, Vlatkovic L, Svindland A (2016). A randomized controlled trial to assess and compare the outcomes of two-core prostate biopsy guided by fused magnetic resonance and transrectal ultrasound images and traditional 12-core systematic biopsy. Eur Urol.

[21] Schoots IG, Roobol MJ, Nieboer D, Bangma CH, Steyerberg EW, Hunink MG (2015). Magnetic resonance imaging-targeted biopsy may enhance the diagnostic accuracy of significant prostate cancer detection compared to standard transrectal ultrasound-guided biopsy: a systematic review and meta-analysis. Eur Urol.

[22] Raventos CX, Orsola A, de Torres I, Cecchini L, Trilla E, Planas J (2010). Preoperative prediction of pathologically insignificant prostate cancer in radical prostatectomy specimens: the role of prostate volume and the number of positive cores. Urol Int.

[23] Helfand BT, Loeb S, Kan D, Catalona WJ (2010). Number of prostate cancer risk alleles may identify possibly ‘insignificant’ disease. BJU Int.

